# Artificial intelligence in oncology publishing: a systematic review and policy analysis of high-impact journals

**DOI:** 10.3389/fonc.2026.1717048

**Published:** 2026-04-01

**Authors:** Raffaele Giusti, Marco Filetti, Pasquale Lombardi, Francesca Lo Bianco, Stefano Sganga, Tommaso Giovagnoli, Giovanni Maria Iannantuono, Andrea Spinazzola, Daniele Santini, Monica Mariniello, Giampiero Porzio, Mohsen Ibrahim, Gennaro Daniele

**Affiliations:** 1 Medical Oncology Unit, Azienda Ospedaliera Sant’Andrea, Rome, Italy; 2 Phase 1 Unit, Agostino Gemelli University Polyclinic (IRCCS), Rome, Italy; 3 Department of Surgery and Cancer, Imperial College London, London, United Kingdom; 4 Department of Medical Oncology A, Universita degli Studi di Roma La Sapienza, Rome, Italy; 5 Tuscany Tumors Association, Home Care Service, Florence, Italy

**Keywords:** artificial intelligence, editorial policy, generative AI, oncology journals, policy analysis, research integrity

## Abstract

**Background:**

Generative artificial intelligence (AI) is reshaping scholarly communication, yet guidance for its responsible use remains uneven across biomedical journals. We aimed to systematically assess editorial policies governing AI-assisted writing in high-impact oncology journals.

**Methods:**

We conducted a systematic review of publicly available editorial and normative documents, operationalized as a cross-sectional policy audit. Oncology journals with a 2023 Journal Impact Factor ≥5 (JCR 2024) were included. Author instructions, editorial policies, and publisher statements issued between January 2020 and March 2025 were analyzed across four domains: authorship, disclosure, permissible uses, and enforcement.

**Results:**

Sixty journals met inclusion criteria. Most journals prohibit AI systems as authors (58/60, 96.7%), reaffirming human accountability. Disclosure of AI use is mandated by 58/60 journals (96.7%), although reporting requirements vary in placement and specificity. Permissible uses are recognized by 58/60 journals (96.7%), generally limited to language editing and formatting under human supervision, while autonomous content generation or interpretation is discouraged. Enforcement provisions are present in 21/60 journals (35.0%), indicating incomplete standardization. At publisher level, disclosure adoption is universal in Elsevier (17/17), Springer Nature (20/20), AACR (6/6), Wiley (6/6), and AMA (1/1), and present in 8/10 journals in the “Other” category. Enforcement varies widely across publishers.

**Discussion:**

Editorial policies show strong convergence on core principles but remain heterogeneous in implementation, particularly regarding enforcement. We propose a cross-publisher “AI Policy Minimum Dataset” including standardized disclosures, defined permissible uses, and proportionate enforcement mechanisms, supported by transparent and regularly updated policy frameworks. Greater harmonization is essential to ensure integrity, accountability, and equitable use of AI in oncology publishing.

## Introduction

The progressive integration of artificial intelligence (AI) technologies within biomedical research has generated a profound transformation in the field of oncology, extending beyond clinical applications and increasingly involving scientific communication and academic publishing. In particular, the advent of large language models (LLMs) has introduced unprecedented opportunities for assisting researchers in the drafting, editing, and refinement of scientific manuscripts (1, 2). These AI-based tools offer valuable support in improving linguistic clarity, correcting grammatical errors, and facilitating the preparation of scholarly texts, especially for authors whose first language is not English (3).

However, alongside these advantages, the use of AI in scientific writing raises crucial concerns regarding authorship, intellectual accountability, and the integrity of the scientific record. A central question within this debate concerns the boundaries between acceptable technical support and the inappropriate delegation of scientific reasoning, interpretation, or creativity to AI systems (4). Recognizing these critical issues, authoritative organizations such as the International Committee of Medical Journal Editors (ICMJE) have established specific recommendations, unequivocally excluding non-human entities from authorship attribution and emphasizing that authorship must be reserved for individuals capable of assuming full responsibility for the accuracy, integrity, and originality of a manuscript’s content (5).

Furthermore, additional guidance has been provided by the Committee on Publication Ethics (COPE) and the World Association of Medical Editors (WAME), which recommend that any use of AI tools in scientific writing must be transparently disclosed, properly documented, and rigorously supervised by human authors (6, 7). Despite these emerging recommendations, significant variability persists among scientific journals and publishers regarding the implementation of AI-related editorial policies, particularly in defining permissible uses, disclosure requirements, and enforcement mechanisms (8).

In the field of oncology, characterized by rapid knowledge translation and direct implications for clinical decision-making, the potential impact of AI-assisted writing on the quality, accuracy, and trustworthiness of scientific literature is particularly relevant (9). In parallel, several major publishing groups—including Springer Nature, Elsevier, and the JAMA Network—have issued centralized policies governing the use of generative AI across their journals, complementing and in some cases superseding individual journal-level instructions (10–12).

To date, however, few studies have systematically examined the editorial policies adopted by oncology journals concerning the use of AI in manuscript preparation. This policy analysis aims to fill this gap by evaluating the current landscape of AI-related editorial policies among the top 60 oncology journals with an Impact Factor (IF) greater than 5, with the objective of mapping existing practices, identifying areas of heterogeneity, and informing the development of harmonized and ethically robust guidelines.

Although this study does not synthesize primary empirical research, it adopts a systematic review framework to examine editorial and normative documents regulating the use of generative AI in scientific writing. Systematic reviews of policy and governance texts are increasingly employed in health research to map regulatory landscapes and identify implementation gaps. Accordingly, we conceptualize this work as a systematic review and policy analysis of editorial practices among high-impact oncology journals.

## Materials and methods

### Study design and objectives

This study was designed as a systematic review of editorial and policy documents, conducted as a cross-sectional audit of AI-related policies among high-impact oncology journals. Rather than synthesizing primary scientific evidence, the review systematically identified, screened, and analyzed publicly available normative texts governing AI-assisted manuscript preparation.

We mapped current editorial practices across four key policy domains:

1. authorship attribution of AI tools;
2. disclosure requirements regarding AI-assisted writing;
3. definition of acceptable uses of AI within manuscript preparation; and
4. presence of enforcement mechanisms and potential sanctions for policy violations

### Journal selection criteria

Eligible journals were identified based on the following inclusion criteria:

- Listed under the Oncology category in the Journal Citation Reports (JCR) 2024
- 2023 Journal Impact Factor ≥ 5.0
- Publication of original research articles in English
- Publicly accessible author instructions and editorial policy documentation in English

A total of 60 oncology journals met these criteria and were included in the final analysis (**Supplementary Table 1**).

### Data sources and collection procedure

Between January 15 and March 15, 2025, a systematic review of each selected journal’s official website was conducted. The search strategy included a comprehensive review of sections dedicated to:

- Instructions for Authors
- Manuscript preparation guidelines
- Editorial policies
- Ethics policies
- Publisher-level AI policy statements

Key terms searched within journal websites included “artificial intelligence,” “AI,” “ChatGPT,” “generative AI,” “authorship,” and “editorial policy.” Instances in which AI-related policy information was absent, ambiguous, or internally inconsistent were coded as “Not Declared.” To resolve ambiguities or inconsistencies identified during the review of publicly available editorial policies, clarification emails were sent to six journals whose policy statements were unclear or internally inconsistent. Two journals provided responses. These clarifications were used exclusively to confirm the interpretation of publicly available policy language and did not introduce additional policy domains beyond those documented in journal instructions. In cases where no response was received, coding was based solely on publicly accessible information.

### Study selection

**Figure 1:**
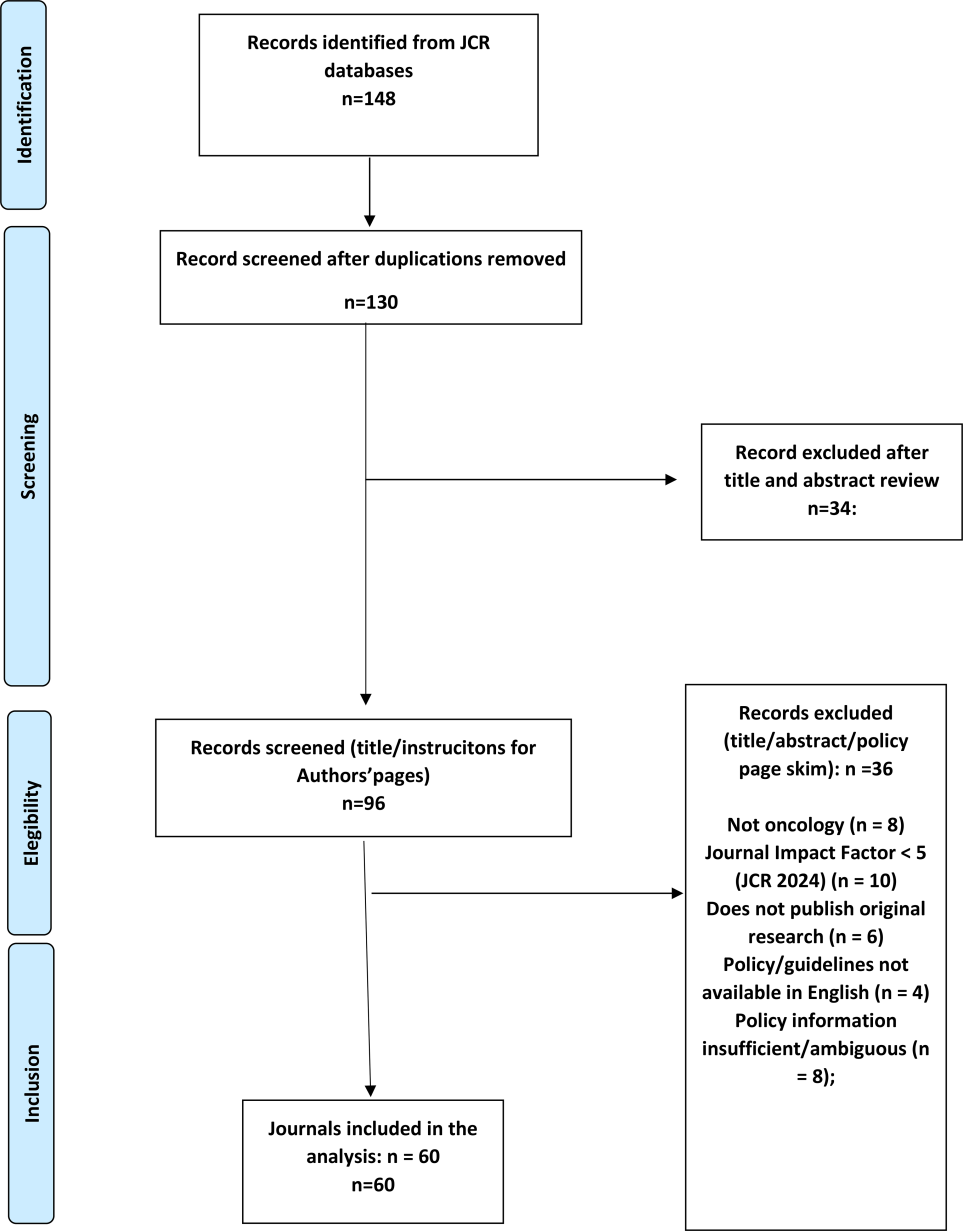
PRISMA 2020 flow diagram adapted for a journal policy audit of oncology journals. Sources: JCR 2024 (Oncology category) and publishers' policy pages; search period 15 Jan- 15 Mar 2025.

Study selection followed the core principles of the PRISMA 2020 framework, adapted for a systematic review of editorial and policy documents rather than primary empirical studies. Out of 148 records initially identified through the JCR database, 130 unique journals remained after deduplication. After title and webpage screening, 96 journals were assessed for eligibility. Thirty-six were excluded based on predefined criteria, leaving 60 journals for full inclusion in the final analysis. This process is summarized in the PRISMA 2020 flow diagram (**Figure 1**).

### Data extraction and coding

Data were collected independently by two investigators (RG and MF) using a standardized data extraction form developed *ad hoc* for this study. Coding was based on a predefined framework developed *a priori*. Initial percent agreement across all policy domains was 96.7%. Discrepancies were resolved through discussion and consensus, with adjudication by a third senior reviewer (PL) when necessary. Given that coding relied on explicit policy statements rather than subjective interpretation, Cohen’s kappa was not calculated. A detailed codebook defining classification criteria for each policy domain is provided as **Supplementary Table 1**.

### Variables of interest

For each journal, the following variables were extracted and recorded:

- Journal name
- 2023 impact factor (JCR 2024)
- Publisher
- Explicit prohibition of AI tools as authors (Yes/No)
- Mandatory disclosure of AI usage (Yes/No)
- Specification of permissible uses of AI (Yes/No; details if available)
- Presence of enforcement policy or sanctions for non-compliance (Yes/No; description if available)

### Data analysis

Collected data were synthesized descriptively and tabulated. Absolute and relative frequencies were calculated for each policy domain. Subgroup analyses were performed to assess differences in policy adoption according to publisher (Elsevier, Springer Nature, Wiley, AACR, AMA, Other). Differences in enforcement adoption across publishers were explored using a chi-square test of independence. Statistical analyses were performed using Microsoft Excel (Version 365). A two-sided p-value < 0.05 was considered statistically significant.

## Results

AI Authorship Prohibition Fifty−eight of 60 journals (96.7%) explicitly prohibit attribution of authorship to AI tools, indicating broad alignment with international authorship guidelines across high−impact oncology publications (Journal IF 2023 ≥ 5; JCR 2024), regardless of publisher or IF (**Table 1**). Two journals lacked explicit posted prohibition.

**Table 1:** summarizes the adoption of key AI-related editorial policy components across the 60 included oncology journals.

Policy domain	Journals adopting policy n (%)
AI authorship explicitly prohibited	58 (96.7%)
Mandatory disclosure of AI use	58 (96.7%)
Permissible uses of AI specified	58 (96.7%)
Enforcement mechanisms or sanctions defined	21 (35.0%)

While near-universal agreement was observed regarding authorship prohibition, disclosure, and permissible AI use, explicit enforcement mechanisms were substantially less frequent, highlighting a marked implementation gap.

AI Disclosure Requirement A mandatory requirement to disclose the use of AI during manuscript preparation was present in 58 of 60 journals (96.7%). Disclosure instructions typically required authors to specify the tool, version, task, and human oversight, though the location of this disclosure varied (e.g., Methods, Acknowledgments, or a dedicated statement). Two journals (3.3%) did not provide explicit guidance on AI−related disclosure. Permissible Uses of AI Fifty−eight journals (96.7%) defined acceptable uses of AI tools, consistently limiting them to non−intellectual tasks such as grammar checking, language polishing, and formatting under transparent human supervision. The use of AI for data interpretation, scientific reasoning, or content generation was uniformly discouraged or explicitly prohibited. Two journals (3.3%) lacked guidance on acceptable AI applications.

Enforcement Mechanisms Enforcement policies specifying consequences for AI−policy violations were present in 21 journals (35.0%). Sanctions included manuscript rejection, retraction, and ethical−misconduct reporting. The majority—39 journals (65.0%)—did not articulate any enforcement measures or post−publication corrective actions.

**Figure 2:**
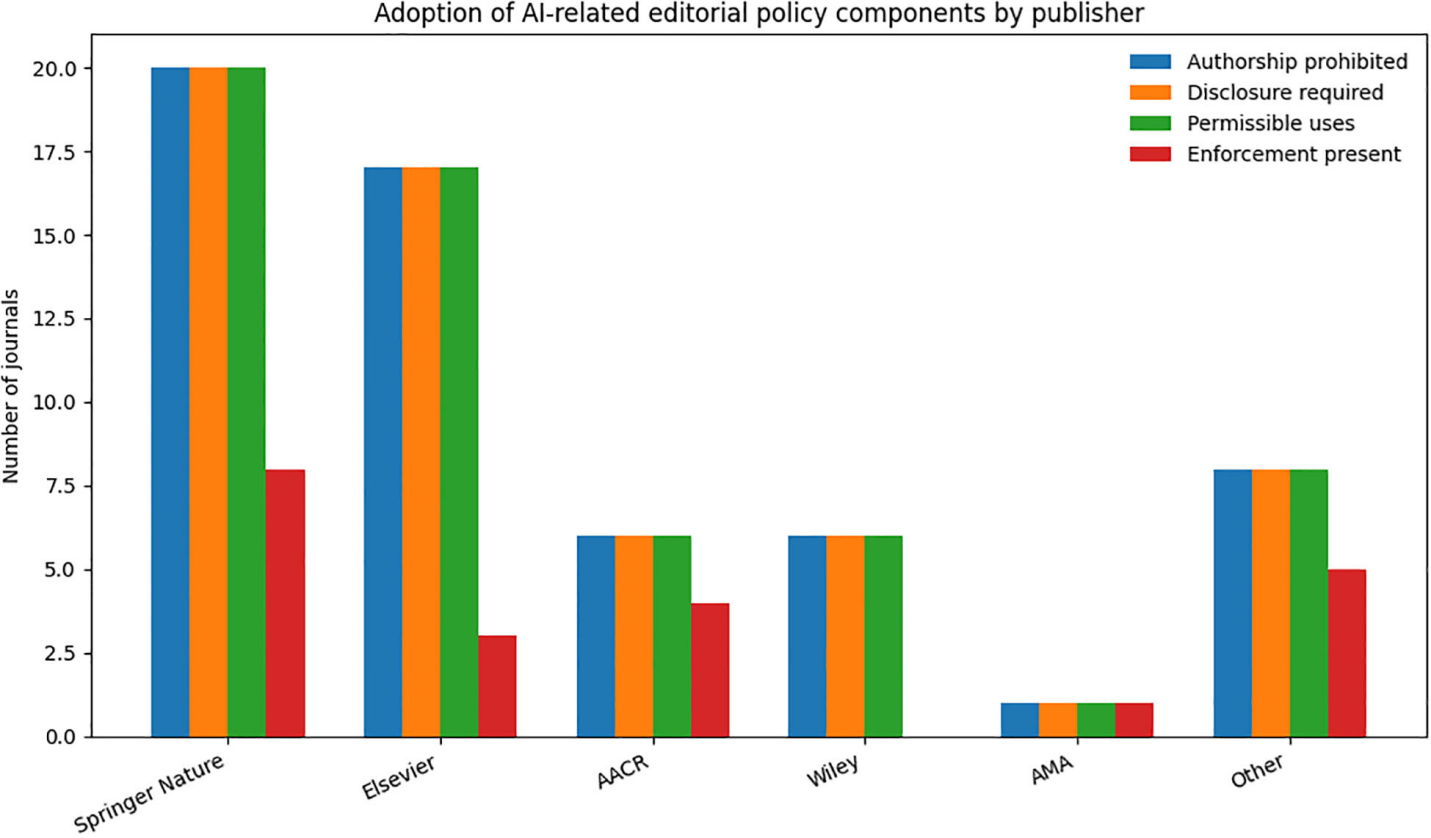
Adoption of AI-related editorial policy components across publishers. Bar chart showing the number of journals implementing four key domains: prohibition of AI authorship, mandatory disclosure of AI use, definition of permissible uses, and presence of enforcement measures. Springer Nature and Elsevier show the highest adoption across domains, while AMA includes fewer journals overall. Enforcement remains the least consistently implemented component across publishers.

Publisher−Specific Policy Distribution Analysis by publishing house shows that the requirement to disclose generative AI use is widespread (58/60, 96.7%). Complete adherence is observed in Elsevier (17/17), Springer Nature (20/20) and AACR (6/6). Among single−title publishers grouped as “Other,” disclosure is present in 8/10 journals; Wiley shows 6/6 (100%) and AMA displays 1/1 (100%). Greater heterogeneity emerges for enforcement mechanisms. Publishers coupling disclosure with explicit sanctions are AACR (4/6, 67%), Springer Nature (8/20, 40%) and Elsevier (3/17, 18%). Wiley specifies 0/6 (0%), AMA 1/1 (100%), and the “Other” group includes 5/10 (50%) journals with explicit post−publication remedies. A chi-square test demonstrated a statistically significant difference in enforcement adoption across publishers (χ² = 11.19, df = 5, p = 0.048), indicating heterogeneous implementation patterns among publishing groups (**Figure 2**).

## Discussion

This systematic review and policy analysis provides one of the first comprehensive mappings of editorial governance regarding generative AI-assisted manuscript preparation in high-impact oncology journals. While recent commentaries and position statements have articulated ethical concerns related to AI use in scientific writing, empirical evaluations of how these principles are translated into journal-level policies remain limited. High-profile editorial commentaries and empirical analyses have emphasized that generative AI may enhance linguistic fluency while simultaneously introducing risks related to accountability, factual accuracy, and trust in the scientific record, reinforcing the need for clear editorial governance (13–15).

Our findings demonstrate substantial convergence on foundational norms—human authorship, transparency, and restricted technical use—alongside marked heterogeneity in enforcement, revealing a critical gap between ethical consensus and practical governance.

The alignment on authorship prohibition reflects international guidance: authorship entails public responsibility and accountability, which non-human tools cannot assume (5, 11). Our data indicates that oncology journals have adopted this standard at scale, extending prior observations from broader biomedical publishing (1, 2). By contrast, disclosure practices remain variably specified—the required placement within the manuscript, the level of reporting specificity (tool, version, functions), and acceptable wording are inconsistently defined—allowing ambiguity and unintentional non-compliance in routine use (6, 7, 10).

Permissible uses are appropriately narrow and technical, consistent with WAME/COPE recommendations that endorse grammar and clarity support under transparent human oversight while discouraging content generation, interpretation, or data analysis by AI without rigorous supervision (6, 7). This equilibrium acknowledges the pragmatic benefits of language assistance—particularly for non-native English-speaking authors—while guarding against fabrication, citation errors, and “hallucinations.” (3, 11).

The consistent restriction of permissible AI uses to linguistic and formatting support is supported by empirical evidence showing that generative language models may produce factually incorrect statements, fabricated references, or misleading interpretations when used autonomously. Experimental studies comparing AI-generated and human-written scientific texts suggest that surface-level fluency may mask substantive inaccuracies that can evade detection during peer review, and that AI-detection tools have important limitations in real-world editorial workflows (2, 4, 8, 9).

The most salient finding of this analysis is the limited articulation of enforcement mechanisms. Despite near-universal endorsement of disclosure and authorship principles, only one third of journals explicitly describe consequences for policy violations. This disconnect mirrors observations from broader analyses of research integrity policies, in which normative expectations are frequently articulated without corresponding procedural safeguards. In the absence of defined enforcement pathways, compliance with AI-related policies relies predominantly on author self-report, raising concerns about consistency, verifiability, and deterrence. We therefore recommend a minimum, field-wide enforcement framework that includes: (1) standardized disclosure text at submission; (2) editorial verification steps during triage; (3) clear taxonomies of breaches tied to proportionate remedies; and (4) post-publication correction pathways (10–12).

From a governance perspective, effective editorial regulation requires alignment between normative standards, operational procedures, and enforcement mechanisms. Policies that articulate ethical principles without specifying verification processes or proportional remedies risk functioning as symbolic statements rather than actionable rules. Our findings suggest that, in the context of AI-assisted writing, many oncology journals remain at an early stage of regulatory maturity, characterized by principle-setting without full procedural integration (16).

Considerations of equity and clarity also support harmonization. Divergent requirements across journals create avoidable administrative friction and complicate multi-journal submissions in fast-moving oncology domains. A cross-publisher consensus—potentially led by editorial consortia and learned societies—could define an “AI Policy Minimum Dataset” comprising a uniform authorship ban, a templated disclosure (tool, version, task, human supervision), a common list of permissible uses, and a shared enforcement lexicon. Such standardization would raise the floor of practice while preserving journal-specific nuances (5–7). The proposed “AI Policy Minimum Dataset” should be interpreted not as a prescriptive or punitive framework, but as a pragmatic governance tool aimed at reducing ambiguity, administrative burden, and unintended non-compliance. Similar minimum datasets and core outcome sets have been implemented in other areas of biomedical research to harmonize reporting standards while preserving contextual flexibility (17, 18).

This study has limitations. First, it relies primarily on publicly accessible editorial and publisher policy documents, which may evolve over time. Second, limited email-based clarifications were used to resolve ambiguities in a subset of journals during a defined time window (January–March 2025); subsequent policy updates may therefore not be captured. Third, occasional internal inconsistencies across different sections of the same journal website were observed, underscoring the need for version-controlled and centralized policy statements. Finally, our sample is restricted to oncology journals with IF ≥ 5, limiting generalizability. Nonetheless, the systematic and transparent approach adopted provides a reproducible baseline against which future policy evolution and real-world compliance can be evaluated.

In summary, oncology journals have coalesced around the twin principles of human authorship and transparent disclosure, but enforcement remains the Achilles’ heel. Prioritizing standardized disclosure templates, explicit enforcement pathways, and regular policy updates will be essential to preserve integrity while enabling responsible innovation in AI-assisted scientific writing (19–24). Future research should examine real world compliance, effects on editorial workflows and peer review efficiency, and the downstream impact on the reliability of oncology literature (1–7, 10–12).

## Conclusions

Oncology journals have largely agreed on the principles for AI use; the task now is to make them work in practice. We recommend a field-wide minimum standard that is simple, transparent, and enforceable: a templated disclosure at submission (with clear reporting specificity), a short list of allowable tasks under human oversight, and a proportionate set of remedies for breaches. These elements should be anchored by stamped, version-controlled policy pages, author attestations, and post-publication correction pathways, with periodic public audits to track uptake and impact. Coordinated updates by publishers, societies, and indexers will keep policies aligned with evolving tools, while attention to language support preserves equity for non-native English-speaking authors.

Without harmonized, enforceable standards, the rapid integration of AI into scientific workflows risks undermining trust in oncology research. Proactive editorial governance will be essential to align innovation with accountability.

## Data Availability

The original contributions presented in the study are included in the article/**Supplementary Material**. Further inquiries can be directed to the corresponding author.
