# Supplementary material for: Artificial intelligence in oncology publishing: a systematic review and policy analysis of high-impact journals
Source: Front Oncol. 2026 Apr 1;16:1717048. doi: 10.3389/fonc.2026.1717048 (PMC13079186; doi:10.3389/fonc.2026.1717048)
Supplement: Supplementary file 1 [file Table1.docx]

# Table S1. AI Policy Components in 60 High-Impact Oncology Journals (Impact Factor 2023; JCR 2024)

| Journal | Publisher | Impact Factor (2023) | AI Authorship Prohibited | AI Disclosure Required | Permissible AI Uses | Enforcement Present |
| --- | --- | --- | --- | --- | --- | --- |
| CA-A Cancer Journal for Clinicians | Wiley | 503.1 | Yes | Not declared | Not declared | Not declared |
| Nature Reviews Clinical Oncology | Springer Nature | 81.1 | Yes | Yes | Yes | Yes |
| Nature Reviews Cancer | Springer Nature | 72.5 | Yes | Yes | Yes | Yes |
| Annals of Oncology | Elsevier | 56.7 | Yes | Yes | Yes | No |
| Cancer Cell | Elsevier (Cell Press) | 48.8 | Yes | Yes | Yes | Yes |
| Journal of Clinical Oncology | ASCO | 42.1 | Yes | Yes | Yes | Yes |
| The Lancet Oncology | Elsevier | 41.6 | Yes | Yes | Yes | Yes |
| Cancer Discovery | AACR | 30.6 | Yes | Yes | Yes | Yes |
| Journal of Hematology & Oncology | Springer Nature | 29.5 | Yes | Yes | Yes | No |
| JAMA Oncology | AMA | 28.4 | Yes | Yes | Yes | Yes |
| Molecular Cancer | Springer Nature | 27.7 | Yes | Yes | Yes | No |
| Nature Cancer | Springer Nature | 23.5 | Yes | Yes | Yes | Yes |
| Cancer Communications | Wiley | 20.1 | Yes | Yes | Yes | No |
| Neuro-Oncology | Oxford University Press | 16.4 | Yes | Yes | Yes | Yes |
| The Lancet Haematology | Elsevier | 15.4 | Yes | Yes | Yes | Yes |
| Journal of the National Comprehensive Cancer Network | Harborside Press | 14.8 | Not declared | Not declared | Not declared | Not declared |
| Trends in Cancer | Elsevier | 14.3 | Yes | Yes | Yes | No |
| JACC: CardioOncology | Elsevier | 13.6 | Yes | Yes | Yes | No |
| Blood Cancer Journal | Springer Nature | 12.9 | Yes | Yes | Yes | No |
| Leukemia | Springer Nature | 12.8 | Yes | Yes | Yes | No |
| Cancer Research | AACR | 12.5 | Yes | Yes | Yes | No |
| Seminars in Cancer Biology | Elsevier | 12.1 | Yes | Yes | Yes | No |
| Blood Cancer Discovery | AACR | 11.7 | Yes | Yes | Yes | No |
| Liver Cancer | Karger | 11.6 | Yes | Yes | Yes | No |
| Journal of Experimental & Clinical Cancer Research | Springer Nature | 11.4 | Yes | Yes | Yes | Yes |
| Clinical Cancer Research | AACR | 10.4 | Yes | Yes | Yes | Yes |
| Journal for Immunotherapy of Cancer | BMJ | 10.3 | Yes | Yes | Yes | Yes |
| Cancer Immunology Research | AACR | 10.1 | Yes | Yes | Yes | Yes |
| JNCI – Journal of the National Cancer Institute | Oxford University Press | 9.9 | Yes | Yes | Yes | Yes |
| BBA – Reviews on Cancer | Elsevier | 9.7 | Yes | Yes | Yes | No |
| Cancer Treatment Reviews | Elsevier | 9.6 | Yes | Yes | Yes | No |
| Biomarker Research | Springer Nature | 9.5 | Yes | Yes | Yes | Yes |
| Experimental Hematology & Oncology | Springer Nature | 9.4 | Yes | Yes | Yes | Yes |
| Cancer Letters | Elsevier | 9.1 | Yes | Yes | Yes | No |
| Clinical and Translational Medicine | Wiley | 7.9 | Yes | Yes | Yes | No |
| Cancer and Metastasis Reviews | Springer Nature | 7.7 | Yes | Yes | Yes | Yes |
| European Journal of Cancer | Elsevier | 7.7 | Yes | Yes | Yes | No |
| Journal of the National Cancer Center | Elsevier | 7.6 | Yes | Yes | Yes | No |
| ESMO Open | Elsevier | 7.1 | Yes | Yes | Yes | No |
| Oncogene | Springer Nature | 6.9 | Yes | Yes | Yes | No |
| npj Precision Oncology | Springer Nature | 6.8 | Yes | Yes | Yes | No |
| npj Breast Cancer | Springer Nature | 6.5 | Yes | Yes | Yes | No |
| OncoImmunology | Taylor & Francis | 6.5 | Yes | Yes | Yes | No |
| British Journal of Cancer | Springer Nature | 6.4 | Yes | Yes | Yes | Yes |
| Neoplasia | Elsevier | 6.3 | Yes | Yes | Yes | No |
| Breast Cancer Research | Springer Nature | 6.1 | Yes | Yes | Yes | No |
| Cancer | Wiley | 6.1 | Yes | Not declared | Not declared | Not declared |
| Gastric Cancer | Springer Nature | 6.0 | Yes | Yes | Yes | No |
| Cancer & Metabolism | Springer Nature | 6.0 | Yes | Yes | Yes | No |
| Oncogenesis | Springer Nature | 5.9 | Yes | Yes | Yes | No |
| Breast | Elsevier | 5.7 | Yes | Yes | Yes | No |
| International Journal of Cancer | Wiley | 5.7 | Yes | Not declared | Not declared | Not declared |
| Cancer Biology & Medicine | CACA | 5.6 | Not declared | Not declared | Not declared | Not declared |
| Critical Reviews in Oncology/Hematology | Elsevier | 5.5 | Yes | Yes | Yes | No |
| Molecular Therapy – Oncolytics | Elsevier | 5.3 | Yes | Yes | Yes | No |
| Molecular Cancer Therapeutics | AACR | 5.3 | Yes | Yes | Yes | Yes |
| Cancer Cell International | Springer Nature | 5.3 | Yes | Yes | Yes | No |
| JCO Precision Oncology | ASCO | 5.3 | Yes | Yes | Yes | Yes |
| Lung Cancer: Targets and Therapy | Dovepress | 5.1 | Yes | Yes | Yes | No |
| Molecular Oncology | Wiley | 5.0 | Yes | Yes | Yes | No |

Legend: Yes = explicitly stated in publicly available editorial policies; No = explicitly excluded; Not declared = absent or ambiguous after systematic review of publicly available documents.
